# Localization of *Helicobacter *spp. in the fundic mucosa of laboratory Beagle dogs: an ultrastructural study

**DOI:** 10.1186/1297-9716-42-42

**Published:** 2011-03-02

**Authors:** Anna Lanzoni, Ivo Faustinelli, Patrizia Cristofori, Mario Luini, Kenneth W Simpson, Eugenio Scanziani, Camilla Recordati

**Affiliations:** 1 Pathology Department, GlaxoSmithKline S.p.A. Medicine Research Centre, 37135 Verona, Italy; 2 Pathology Department, GlaxoSmithKline R&D, Park Road SG120DP, Ware, Herts, UK; 3 Istituto Zooprofilattico Sperimentale della Lombardia e dell'Emilia Romagna, Sezione di Lodi, 26900 Lodi, Italy; 4 Department of Clinical Sciences, College of Veterinary Medicine, Cornell University, Ithaca, NY 14853, USA; 5 Dipartimento di Patologia Animale, Igiene e Sanità Pubblica Veterinaria, Facoltà di Medicina Veterinaria, Università degli Studi di Milano, 20133 Milano, Italy; 6 Mouse and Animal Pathology Laboratory, Fondazione Filarete, 20139 Milano, Italy

## Abstract

In dogs *Helicobacter *spp. are found in all gastric regions usually localized in the surface mucus, gastric glands and parietal cells. The aim of this study was to detail the distribution of *Helicobacter *spp. in the fundic mucosa of asymptomatic Beagle dogs and their intracellular localization within parietal cells, in order to evaluate species-specific pathogenetic effects on gastric cells. The presence of *Helicobacter *spp. was investigated by immunohistochemistry, TEM, and PCR in the fundic mucosa of six Beagle dogs. *Helicobacter *spp. were found in all dogs examined, and *H. bizzozeronii *and *H. felis *were identified by PCR and confirmed by TEM. In the lumen of the fundic glands, co-localization was common. *H. bizzozeronii *was present in larger numbers than *H. felis *in both intraluminal and intraparietal localization. The amounts of *H. bizzozeronii *were similar in superficial and basal portions of the glands. *H. felis *was predominantly localized in the superficial portions of gastric glands but almost absent from the base. Within parietal cells, most *Helicobacter *organisms were intracanalicular, but intact and degenerate *Helicobacter *organisms were also visualized free in the cytoplasm or in secondary lysosomes. No specific degenerative lesions were found in infected parietal cells. *Helicobacter *organisms were also observed within macrophages in the lamina propria. In conclusion, there is a differential distribution of *H. bizzozeronii *and *H. felis *in the fundic mucosa of Beagle dogs, and their intracellular localization in parietal cells and macrophages suggests novel pathogenic scenarios for the development of immune response and maintenance of chronic gastritis in dogs.

## Introduction

Gastric colonization by spiral-shaped bacteria of the genus *Helicobacter *is common in dogs and to date several species of *Helicobacter *have been identified in the canine stomach, including *Helicobacter felis*, *Helicobacter bizzozeronii*, *Helicobacter salomonis*, "*Candidatus *Helicobacter heilmannii", *Helicobacter bilis*, *Helicobacter *(*Flexispira*) *rappini*, and *Helicobacter cynogastricus *[1-6]. Dogs may be infected with more than one species of *Helicobacter *that are indistinguishable on light microscopy, appearing as large tightly coiled spiral-shaped bacteria [7]. Different species of canine gastric *Helicobacter *can be categorized by their morphological features (i.e. size, number and degree of tightness of the spirals, presence of periplasmic fibrils, number and position of flagella) using transmission electron microscopy (TEM). In addition, in the early work of Lockard and Boler (1970) morphologically different organisms were classified as Lockard types 1 to 3 [8,9].

The regional distribution of *Helicobacter *spp. throughout the canine stomach (i.e. cardia, fundus, and pylorus) is well studied [7], but knowledge of the distribution of different *Helicobacter *spp. within these regions is incomplete. *Helicobacter *organisms in dogs reside in the surface mucus, gastric pits, and gastric glands, colonizing both the lumen and the gastric cells. In particular, canine and feline *Helicobacter *spp. (as "*Candidatus *H. heilmannii" in humans) have great predilection for parietal cells of the fundic mucosa, where they are usually described to colonize the intracellular canaliculi and sporadically the cytoplasm [7,10-14]. Since the virulence of many pathogenic bacteria is directly related to invasion it is possible that the development of *Helicobacter *associated gastritis in dogs is related to their intracellular localization (intracanalicular or intracytoplasmic) or possibly invasion of the lamina propria through the basal lamina, as reported for *H. pylori*, which is now considered as an invasive and facultative intracellular organism [15-17]. The ability of *Helicobacter *spp. infection in dogs to establish an intracellular niche may also vary by species or even strain, and this may impact on their pathogenicity and ability to evade antibiotics typically employed to treat *Helicobacter *gastritis in dogs [18].

The aim of this study was to gain insight into the distribution and localization of distinct *Helicobacter *spp. in the lumen of gastric glands and within parietal cells in the fundic mucosa, using a limited number of asymptomatic Beagle dogs. This was done by applying a combination of morphological (immunohistochemical and ultrastructural) and molecular methods (species-specific PCR) and allowed to evaluate the presence of distinct colonization niches and possible species-specific pathogenetic effects on gastric cells.

## Materials and methods

### Animals and sampling

Three male (No. 1, 2, 3) and three female (No. 4, 5, 6) Beagle dogs (Marshall Beagles), purchased from Greenhill Italy (Montichiari, Brescia, Italy) were investigated. The dogs were 8-9 months old, and were part of a general toxicology study performed in GlaxoSmithKline S.p.A., where they served as the control group. They were housed singly or in pairs, in standard husbandry and environmental conditions. All the work involving animals was carried out in accordance with Italian regulation governing animal welfare and protection (Legislative Decree n. 116/1992), the European Directive 86/609/EEC, and according to the internal GlaxoSmithKline Committee on Animal Research & Ethics (CARE) review, with related codes of practice.

Dogs were euthanized by intravenous injection of barbiturate. Prior to necropsy, the animals were deprived of food overnight. The stomach was opened along the inner curvature and macroscopically examined. Three samples were taken from the body (greater curvature) region from each dog: one was snap frozen in liquid nitrogen and stored at -80°C for PCR analysis; one was fixed in 10% buffered formalin for histopathology; one was fixed in 2.5% glutaraldehyde in 0.1 mol/L cacodylate buffer pH 7.2, containing 0.01 mol/L CaCl_2 _for at least 12 h at 4°C for ultrastructural examination.

### DNA Extraction and PCR amplification

DNA was extracted using DNeasy Tissue kit (QIAGEN, GmbH D-40724, Hilden, Germany) according to the manufacturer's instructions. Primers used in this study for the amplification of *Helicobacteraceae*, canine gastric *Helicobacter *spp. (*H. bizzozeronii*, *H. salomonis *and *H. felis*), canine enterohepatic *Helicobacter *spp., *H. pylori*, "*Candidatus *H. heilmannii", *H. felis*, and *H. bizzozzeronii *are listed in Table 1.

**Table 1:** Oligonucleotide primers used in this study.

Primer	Sequence	Target sequence	Reference
C97	5'- GCT ATG ACG GGT ATC C-3'	16S rRNA gene *Helicobacteraceae*	[19]
C05	5'- ACT TCA CCC CAG TCG CTG-3'	16S rRNA gene *Helicobacteraceae*	[19]
CAR557f	5'- TGC GTA GGC GGG GTT GTA AG-3'	16S rRNA gene *H. felis*, *H. bizzozeronii*, *H. salomonis*	[20]
CAR636r	5'- CAG AGT TGT AGT TTC AAA TGC-3'	16S rRNA gene *H. felis*, *H. bizzozeronii*, *H. salomonis*	[20]
H. pylori 1	5'-GGA ATT CCA GAT CTA TGA AAA AGA TTA GCA GAA AAG-3'	Urease B gene *H. pylori*	[21]
H. pylori 2	5'- GGA ATT CGT CGA CCT AGA AAA TGC TAA AGA GTT G-3'	Urease B gene *H. pylori*	[21]
Heil 1F	5'-GGG CGA TAA AGT GCG CTT G-3'	Urease B gene "*Candidatus *H. heilmannii"	[21]
Heil 2R	5'-CTG GTC AAT GAG AGC AGG-3'	Urease B gene "*Candidatus *H. heilmannii"	[21]
H. felis 1	5'- ATG AAA CTA ACG CCT AAA GAA CTA G-3'	Urease B gene *H. felis*	[21]
H. felis 2	5'-GGA GAG ATA AAG TGA ATA TGC GT-3'	Urease B gene *H. felis*	[21]
H. bizz 1	5'-GAA GTC GAA CAT GAC TGC AC-3'	Urease B gene *H. bizzozeronii*	[5]
H. bizz 2	5'-GGT CGC ATT AGT CCC ATC AG-	Urease B gene *H. bizzozeronii*	[5]
HelINTF1	5'- TGA ATG CTA GTT GTT GCC CTG CTT G-3'	16S rRNA gene emterohepatic *Helicobacter *spp.	[22]
HelINTR1	5'- TCT CCT TAG AGT GCT CAG CCG AAC T-3'	16S rRNA gene enterohepatic *Helicobacter *spp.	[22]

All PCR reactions were performed in an Eppendorf thermocycler (Mastercycler gradient, Eppendorf GA, Hamburg, Germany) in a final volume of 25 μL containing 1 μL of extracted DNA, 12.5 μL of PCR Master Mix (Promega, Madison, Wisconsin, USA), and 0.5 μM of each primer (Integrated DNA Technologies, Coralville, IA, USA). Ten microliters of each amplification product were analyzed by gel electrophoresis in 1% agarose gels in Tris-acetate EDTA buffer (TAE). The gel was stained with ethidium bromide (0.5 mg/L) and examined under UV transilluminator for the presence of amplified DNA; the size of the expected fragment was compared to a 100 bp reference marker (Fermentas Inc, Maryland, USA). Negative controls in which the DNA extract was omitted were included with each reaction.

#### Helicobacteraceae PCR

The 16S rRNA gene of members of the *Helicobacteraceae *family was amplified by PCR using primers C97/C05 [19] generating a 1200 bp amplicon. For C97/C05 amplification samples were heated to 94°C for 10 min, followed by 30 amplification cycles of denaturation at 94°C for 1 min, primer annealing at 58°C for 1 min and 30 s, and extension at 72°C for 2 min, with a final extension at 72°C for 10 min.

#### Gastric Helicobacter spp. PCR

Primers CAR557f and CAR636r were used to amplify a 78 bp fragment of the 16S rRNA gene of the canine gastric *Helicobacter *species (*H. bizzozeronii*, *H. salomonis *and *H. felis*) [20]. For CAR557f/CAR636r amplification samples were heated to 94°C for 9 min, followed by 35 amplification cycles of denaturation at 94°C for 30 s, primer annealing at 60°C for 30 s, and extension at 72°C for 45 s, with a final extension at 72°C for 5 min. Additionally, all gastric samples were investigated using species-specific primers for *H. pylori*, *"Candidatus *H. heilmannii", *H. felis*, and *H. bizzozzeronii *urease B gene amplification, as previously reported [5,21].

#### Enterohepatic Helicobacter spp. PCR

Primers HelINTF1 and HelINTR1 were used to amplify a 345 bp fragment of the 16S rRNA gene of enterohepatic *Helicobacter *spp. [22]. For HelINTF1/HelINTR1 amplification samples were heated to 94°C for 2 min, followed by 27 amplification cycles of denaturation at 94°C for 30 s, primer annealing at 60°C for 30 s, and extension at 72°C for 30 s, with a final extension at 72°C for 7 min.

### Histopathology

For light microscopy examination, formalin-fixed samples were embedded in paraffin wax, sectioned at 4 μm thickness, and stained with hematoxylin and eosin (HE).

For immunohistochemistry (IHC), formalin-fixed, paraffin-embedded 4 μm sections were deparaffinized, rehydrated and treated with 3% hydrogen peroxide in distilled water for 20 min. The sections were labelled by the avidin-biotin-peroxidase (ABC) procedure [23] with a commercial immunoperoxidase kit (Vectastain Standard Elite; Vector Laboratories, Burlingame, CA, USA). A polyclonal rabbit anti-*H. pylori *antibody (Dako, Glostrup, Denmark) was applied at a working dilution of 1:1000 at 4°C overnight. The immunohistochemical reaction was developed with 3,3' diaminobenzidine (DAB) for 1 min and sections were counterstained for 1 min with Mayer's haematoxylin. Negative controls for each sample were prepared by replacing the primary antibody with PBS containing 10% normal goat serum. Known positive control sections were included in each immunolabeling assay.

Samples were examined for the presence of inflammation (infiltrates of lymphocytes, plasma cells, and macrophages) using a semiquantitative scoring system as follows: 0 (absent), +1 (mild), +2 (moderate), +3 (marked).

Immunostained sections were scored for the presence of *Helicobacter *antigen within the parietal cells (P) and in the lumen (L) of gastric glands using a semiquantitative scoring system as follows: 0 (absence of *Helicobacter *antigen in the parietal cell/lumen of the gastric glands); +1 (presence of *Helicobacter *antigen in the parietal cell/lumen of < 5% of the gastric glands); +2 (presence of *Helicobacter *antigen in the parietal cell/lumen of 5-50% of the gastric glands); +3 (presence of *Helicobacter *antigen in the parietal cell/lumen of > 50% of the gastric glands); +4 (presence of abundant *Helicobacter *antigen in the parietal cell/lumen of > 50% of the gastric glands and extending to the base of the glands).

### Transmission electron microscopy (TEM)

Glutaraldehyde fixed samples were transferred into phosphate buffer after 72 h, postfixed for 90 min in 1% osmium tetroxide (wt/vol), and then stained with 2% (wt/vol) uranyl acetate in barbiturate buffer pH 7.3 for 60 min. After dehydration and embedding in Epon (glycide ether 100, Merck, Darmstadt, Germany), 1 μm-thick sections were stained with toluidine blue and the areas of interest were selected. Thin sections were stained with uranyl acetate and lead citrate. Electron microscopy was carried out using a Philips Morgagni™ 268D transmission electron microscope (FEI Company, Eindhoven, The Netherlands) operating at 90 kV.

*Helicobacter *spp. were identified according to their morphological features (size, number of spirals, presence of periplasmic fibrils, number and position of flagella) [2,3,24]. The distribution and number (bacterial load) of *Helicobacter *spp. was evaluated in 10-20 gastric glands and in 4-8 parietal cells of the fundic mucosa from 3 dogs (No. 1-3). The superficial (pit/isthmus/neck) and basal portions of the gastric glands were investigated as distinct colonization sites and the number of distinct morphological types of *Helicobacter *spp. was evaluated within parietal cells (including intracanalicular and intracytoplasmic compartments).

### Statistical analysis

Differences in regional distribution of intraluminal *Helicobacter *load between superficial (pit/isthmus/neck) and basal portions of the gastric glands were evaluated using the Mann-Whitney test. Differences in *Helicobacter *species (*H. bizzozeronii*-like or *H. felis*-like organisms) load in the same location (superficial and basal portions of the gastric glands, and intraparietal localization) were evaluated using the Wilcoxon signed-rank test. Statistical significance was set at *P *= 0.001.

## Results

### PCR

*Helicobacteraceae *and canine gastric *Helicobacter *spp. DNA was amplified in all fundic samples from all six dogs. Species-specific *Helicobacter *PCR detected *H. bizzozeronii *and *H. felis *in all six dogs. PCR for "*Candidatus *H. heilmannii", *H. pylori *and enterohepatic *Helicobacter *spp. was negative for all samples.

### Gross pathology and histopathology

No gross lesions were observed in the stomach of the dogs. Histologically, mild chronic inflammation was present in the fundic mucosa of 5 of 6 dogs (Table 2). Focal to multifocal infiltrates of small numbers of lymphocytes, plasma cells, and macrophages were found in the lamina propria, mainly localized in the deepest portion of the mucosa, close to the *muscularis mucosae*. In HE-stained sections, ill-defined elongated bacteria consistent with *Helicobacter *spp. were visible at high power magnification (400×) in the mucus covering the surface epithelium, the gastric pits, lumen of gastric glands, and occasionally in parietal cells. In all dogs, most parietal cells were morphologically normal; however, small numbers of parietal cells with a large cytoplasmic vacuole containing bacterial organisms were found, localized mainly in the upper third of the gastric gland, around the isthmus region.

**Table 2:** Gastric inflammation and *Helicobacter *spp. colonization of fundic mucosa in 6 laboratory Beagle dogs infected by *Helicobacter *spp.

**Dog no**.	Inflammation (0-3)	*Helicobacter *spp. colonization (IHC) (0-4)	
		
		L	P
1	0	3	3
2	1	4	4
3	1	3	3
4	1	4	4
5	1	3	3
6	1	3	3

L = lumen of gastric glands

P = parietal cells

**Figure 1:**
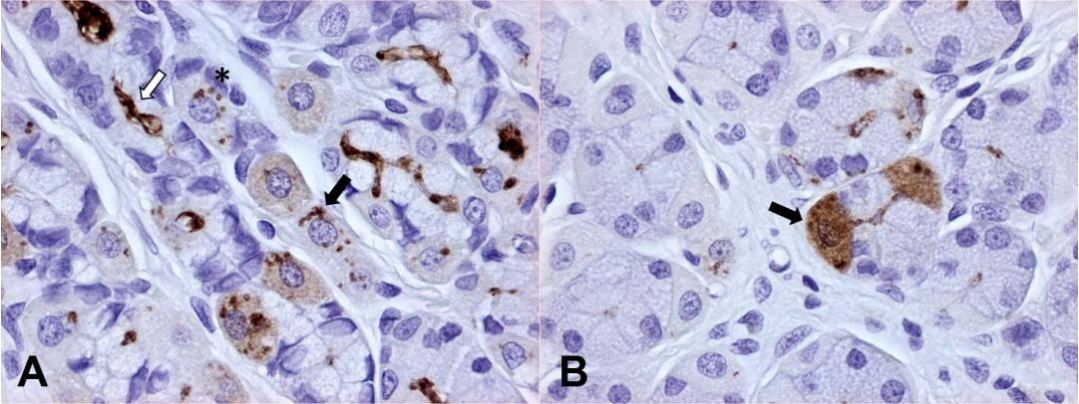
**Dog, fundic mucosa (immunoperoxidase staining, anti-*H. pylori *IHC, 400×)**. Figure 1A - Large amounts of *Helicobacter *antigen in the lumen of fundic glands (white arrow) and within parietal cells, where *Helicobacter *antigen is detectable as well preserved spiral-shaped organisms (black arrow) or brown round dots (asterisk). In scattered parietal cells, a diffuse light cytoplasmic staining is also visible. Figure 1B - *Helicobacter *antigen is occasionally detectable as diffuse and marked cytoplasmic staining (black arrow).

The degree of *Helicobacter *spp. colonization of the stomach assessed by IHC is summarized in Table 2. Positive immunostaining of *Helicobacter *antigen was characterized by a coarse granular brown precipitate easily detectable against the light blue background. Moderate to large numbers of *Helicobacter *spp. were visible in the fundic mucosa, in particular the *Helicobacter *antigen was found in the mucus covering the surface epithelium, the gastric pits, lumen of gastric glands, and intracellularly within parietal cells, multifocally contained in large vacuoles. There was no difference in *Helicobacter *colonization density between the lumen and parietal cells. Within parietal cells, the *Helicobacter *antigen was visible as 1-2 μm in diameter, well demarcated, brown dots or less frequently as a fine brown granular staining diffuse to the whole cytoplasm. Occasionally, well defined spiral shaped organisms were visible in parietal cells (Figure 1).

### Transmission electron microscopy (TEM)

**Figure 2:**
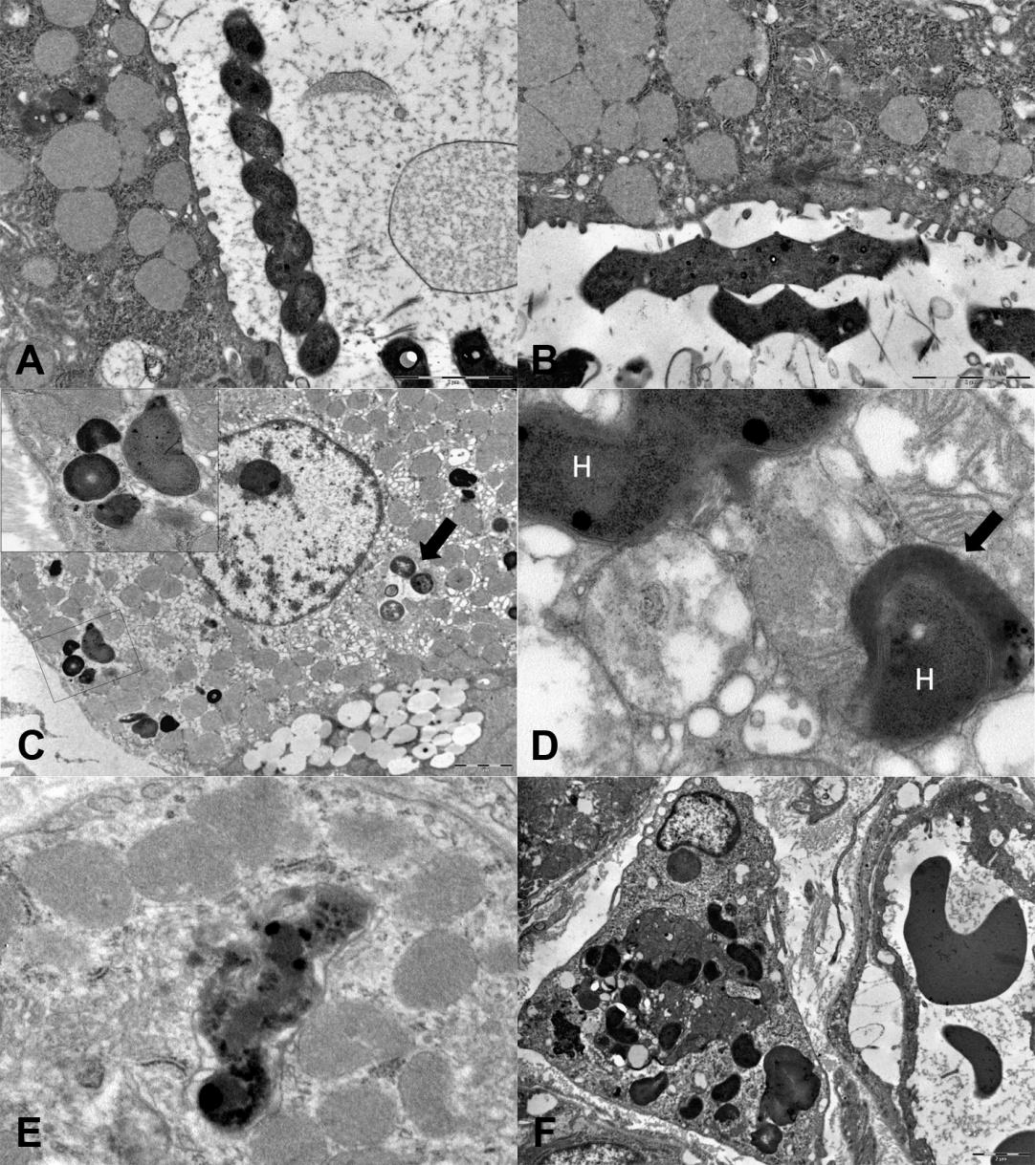
**Dog, ultrastructural investigation of the fundic mucosa**. Figure 2A. Intraluminal *H. bizzozeronii*-like organism. A tightly coiled spiral-shaped organism without periplasmic fibrils is free-floating in the superficial mucus (TEM, original magnification 8900×). Figure 2B. Intraluminal *H. felis*-like organism. A tightly coiled spiral-shaped bacteria with periplasmic fibrils is visible in the superficial mucus; multifocal contact between *H. felis*-like organism and parietal cell microvilli is present (TEM, original magnification 8900×). Figure 2C. Parietal cell containing *Helicobacter*-like organisms. Multiple sections of *Helicobacter*-like organisms were localized within a canaliculus (black arrow) (TEM, original magnification 4 400×). Inset: other bacterial sections were unclearly delimited by parietal cell membranes and adjacent to secondary lysosomes (TEM, original magnification 28000×). Figure 2D. High magnification of the cytoplasm of a parietal cell containing two electrondense intracytoplasmic *Helicobacter*-like organisms (H). In the upper left corner a longitudinal section of an *Helicobacter*-like organism is surrounded by closely apposed cell membranes which are focally interrupted; in the lower left corner a transverse section of *Helicobacter*-like organism is partially enclosed in a lysosome (black arrow) (TEM, original magnification 28000×). Figure 2E. Parietal cell with degenerate *Helicobacter*-like organism characterized by fragmentation and condensation of bacterial contents and dispersion into the parietal cell cytosol (TEM, original magnification 14000×). Figure 2F. Lamina propria of the fundic mucosa. Macrophage in the interstitium between a blood vessel (right) and the basal portion of gastric epithelial cells resting on a basal lamina (upper left corner) containing multiple profiles of well preserved intracytoplasmic *Helicobacter*-like organisms and electrondense remnants of an entire phagocytized cell, most likely a parietal cell (TEM, original magnification 8900×).

Bacteria were identified by TEM in the fundic mucosa from all the dogs examined. They appeared as numerous, 4-8 μm in length × 0.6-0.8 μm in width, tightly to loosely coiled spiral-shaped bacteria with or without periplasmic fibrils, and with bipolar flagellar tufts (composed of up to six flagella) consistent with two types of large canine gastric *Helicobacter *spp.. According to the results of PCR and aforementioned ultrastuctural features they were identified as *H. bizzozeronii*-like and *H. felis*-like (Figures 2A and 2B).

In the superficial mucus layer, low numbers of free-floating *Helicobacter *spp. were seen, occasionally admixed with bacteria with disrupted walls and variably condensed or vacuolated cytoplasm (degenerated bacteria). In the lumen of the fundic glands, co-localization of the two morphologically distinct *Helicobacter *spp. was common. *Helicobacter *spp. load in the lumen of gastric glands and parietal cells is summarized in Table 3. Intraluminal load of total *Helicobacter*-like organisms and *H. bizzozeronii*-like organisms was similar in superficial and basal portions of the glands (*P *= 0.4146 and *P *= 0.4817), while the load of *H. felis*-like was significantly higher in the superficial portions compared to the base (*P *= 0.0007). Both in the superficial and basal portions, there were significantly more *H. bizzozeronii*-like organisms than *H. felis*-like organisms (*P *< 0.0001). In parietal cells, there were significantly more *H. bizzozeronii-*like organisms than *H. felis*-like organisms (*P *= 0.0004).

**Table 3:** *Helicobacter *spp. load in gastric glands and parietal cells evaluated by electron microscopy

	Intraluminal localization					Intracellular localization			
	
**Dog No**.	Gastric gland portion	No. of fundic glands	*Helicobacter*-like organisms mean (SD)	*H. bizzozeronii*-like organism mean (SD)	*H. felis*-like organism mean (SD)	No. of parietal cells	*Helicobacter*-like organisms per cell mean (SD)	*H. bizzozeronii-*like organisms per cell mean (SD)	*H. felis*-like organisms per cell mean (SD)
1	Pit/isthmus/neck	15	3.33 (± 2.06)	1.80 (± 1.57)	1.53 (± 1.46)	5	2.40 (± 1.14)	2.20 (± 1.10)	0.20 (± 0.45)
	Base	11	3.27 (± 2.00)	3.09 (± 1.97)	0.18 (± 0.40)				

2	Pit/isthmus/neck	15	2.53 (± 1.06)	1.73 (± 1.22)	0.80 (± 1.21)	8	1.63 (± 0.74)	1.50 (± 0.76)	0.13 (± 0.35)
	Base	12	2.92 (± 1.65)	2.67 (± 1.67)	0.25 (± 0.45)				

3	Pit/isthmus/neck	20	4.75 (± 2.61)	4.70 (± 2.49)	0.05 (± 0.22)	4	2.75 (± 2.36)	2.75 (± 2.36)	0.00 (± 0.00)
	Base	10	3.40 (± 1.96)	3.40 (± 1.96)	0.00 (± 0.00)				

Total	Pit/isthmus/neck	50	3.66 (± 2.26)	2.94 (± 2.38)	0.72 (± 1.20)	17	2.12 (± 1.36)	2.00 (± 1.37)	0.12 (± 0.33)
	Base	33	3.18 (± 1.79)	3.03 (± 1.83)	0.15 (± 0.36)				

Intraluminal bacteria were localized close to the luminal surface of gastric epithelial cells and the luminal pole of the intracanalicular system of parietal cells. Although *H. felis*-like organisms were often in contact with parietal cell membranes and microvilli, no adhesion to cellular membranes, pedestal formation, actin condensation or disruption of intercellular junctions were found. Within parietal cells, electrondense structures morphologically consistent with bacterial organisms were frequently identified. In most cases, bacteria were found in the intracellular portion of the canalicular system (Figure 2C). Both well preserved spiral-shaped forms, with or without periplasmic fibrils, and rounded bodies (transverse sections of bacteria) were observed (Figure 2C). Bacteria were also present within the cytoplasm of parietal cells enveloped by a closely apposed membrane, occasionally focally disrupted, or free in the cytoplasm or within secondary lysosomes (Figure 2D). Condensation or lysis of bacterial structures consistent with bacterial degeneration was occasionally visualized within the cytoplasm of parietal cells (Figure 2E). In both colonized and uncolonized parietal cells, secondary lysosomes were often present and degenerating swollen mitochondria were occasionally observed. Macrophages in the lamina propria of fundic mucosa had numerous secondary lysosomes similar to those detected in parietal cells, and, rarely, intracellular *Helicobacter *organisms were found in their cytoplasm. In a single case, *Helicobacter *organisms were in association with remnants of an entire phagocytized cell (Figure 2F).

## Discussion

*Helicobacter *spp. were identified by histology, IHC, TEM, and PCR in the fundic mucosa of all the dogs examined in this study, confirming the high prevalence (100%) of the infection in healthy laboratory Beagle dogs [1,12,25]. *Helicobacter*-like organisms were visualized in the surface mucus, lumen of gastric glands (extending from the gastric pit to the base), and within parietal cells, but not within mucous epithelial cells and chief cells, in agreement with previous studies [12,13,26]. On the basis of species-specific PCR, all of the dogs were found to be coinfected with *H. bizzozeronii *and *H. felis *and this finding was supported by TEM, which showed two morphologically distinct *Helicobacter *types, *H. bizzozeronii*-like and *H. felis*-like, in the fundic mucosa. Coinfection with *H. bizzozeronii*-like and *H. felis*-like organisms, has been previously observed in laboratory Beagles [12,25].

In depth analysis of TEM images suggests that these *Helicobacter *species are adapted for colonization of distinct niches in the fundic mucosa. In the lumen of fundic glands, co-localization of the two distinct morphologic types was common. *H. bizzozeronii-*like organisms colonized the glands from the pit to the base, while *H. felis*-like organisms were localized in the more superficial portions of the gastric glands (pit, isthmus and neck regions), but virtually absent from the base. In all dogs and portions of gastric glands evaluated, *H. bizzozeronii*-like organisms were significantly more abundant than *H. felis*-like organisms. Within parietal cells, *H. bizzozeronnii *was also the most common species, and this can be simply the result of the lower abundance of *H. felis *in the glands of these dogs or a consequence of the lower affinity of *H. felis *towards the parietal cells compared to *H. bizzozeronii*. The latter hypothesis is supported by the presence of higher proportion of *H. felis*-like organisms in the glands versus parietal cells (*H. felis *in glands/parietal cells = 4.05) compared to *H. bizzozeronii*-like organisms (*H. bizzozeronii *in glands/parietal cells = 1.49). Altogether, these findings may indicate that *H. bizzozeronii *could be more host-adapted then *H. felis *in dogs.

Some previous studies have suggested that *H. felis *is more pathogenic than *H. bizzozeronii *in dogs and also experimentally in mice and gerbils, resulting in necrosis and/or apoptosis of parietal cells with subsequent parietal cell loss, whereas *H. bizzozeronii *appears non pathogenic even in cases of heavy bacterial load [27-30]. In the present study, however, there were no specific and relevant ultrastructural parietal cell degenerative lesions with either of *Helicobacter *species, but the lack of cell damage could also be related to the limited sampling of the fundic mucosa that was performed in this study. Additionally, no evidence of tight attachment to gastric epithelial cells and no intercellular invasion by canine gastric *Helicobacter *spp. were found and this contrasts with *H. pylori *which is commonly found to attach to the cell membrane of epithelial cells by different forms of adhesive structures (i.e. pedestals or membrane fusion), and invade intercellular space with disruption of intercellular junctions [15,17,31]. Although there was no attachment to the cell membrane, *H. felis*-like organisms were often in direct but loose contact with the plasma cell membranes and microvilli of parietal cells, confirming previous ultrastructural observations performed in dogs and gerbils [27,30].

Canine and feline *Helicobacter *spp., as well as "*Candidatus *H. heilmannii" in humans, have greater affinity to parietal cells than does *H. pylori *in man, and are usually found in their intracellular canaliculi [7,10-13,27,32,33]. Also in the present study most of the intracellular organisms were intracanalicular, but cross-sections of *Helicobacter *organisms or degenerating bacterial fragments were found directly free in the cytoplasm of parietal cells, in association with secondary lysosome-like structures, or occasionally enveloped by a closely apposed membrane which could be focally interrupted resulting in the partial penetration of the organism into the cytoplasm. These ultrastructural findings are consistent with a true intracytoplasmic localization, often associated with bacterial degradation. It is not clear how *Helicobacter *organisms penetrate into the cytoplasm of parietal cells, and whether the intracytoplasmic organisms are associated with parietal cell degeneration, since only sporadic degenerative alterations of organelles were found in both infected and uninfected parietal cells.

By IHC, intracellular *Helicobacter *antigen within parietal cells appeared as well demarcated dots or diffuse cytoplasmic staining of parietal cells. Based on the ultrastructural findings, the immunohistochemical dots are likely the cross-sections of intracellular bacteria or their degrading forms, while the diffuse cytoplasmic staining could be the result of cytoplasmic bacterial degradation and dispersion of bacterial contents (including antigens) throughout the cytoplasm.

Sporadically, *Helicobacter *spp. and their fragments were found by TEM, but not by IHC, in the cytoplasm of macrophages in the lamina propria of the fundic mucosa. The presence of *Helicobacter *organisms within macrophages can be the result of phagocytosis of bacteria released in the extracellular space after parietal cell necrosis or phagocytosis of degenerated parietal cells containing intracellular bacteria, as suggested by the presence of intracytoplasmic remnants of an entire phagocytized cell in association with *Helicobacter *organisms within a macrophage found in the lamina propria. Direct invasion of lamina propria with phagocytosis by macrophages seems less likely the mechanism since no evidence of transepithelial invasion was found. To our knowledge, canine *Helicobacter *spp. have not been so far described within macrophages, in contrast to *H. pylori *which has been described to invade the lamina propria and translocate via macrophages to gastric lymph nodes, leading to the chronic stimulation of the immune system and thus contributing to the maintenance of chronic gastritis in people [15-17,34].

In the fundic mucosa of this small group of Beagle dogs, we observed high levels of mucosal *Helicobacter *colonization but clinical signs of gastritis were absent and only mild chronic lymphoplasmacytic gastritis was detected. The absence of a correlation between *Helicobacter *colonization density, severity of inflammation, and clinical signs has been reported by several studies [7,13,14,26,35].

Previous studies have demonstrated that *Helicobacter *infection in dogs induces a specific systemic humoral response with reported seroconversion after infection and a Th1-biased local immune response [14,27,35], but the mechanisms of activation of the immune response in association with canine *Helicobacter *spp. infection have been so far not investigated. The intracytoplasmic localization of canine *Helicobacter *spp. in parietal cells and macrophages in the lamina propria disclose novel pathogenic scenarios for the development of the cell-mediated and humoral immune response and maintenance of chronic gastritis in dogs. Activation of the immune response might be related to an antigenic stimulation of CD4+ T cells either by direct epithelial cell presentation of *Helicobacter *antigens in combination with induced expression of class II MHC and B7 costimulatory molecules, or by lamina propria macrophages after phagocytosis of *Helicobacter *spp. secondary to parietal cell necrosis, with degradation and antigen presentation directly to immunocompetent cells recruited in the lamina propria or after nodal migration, similarly to what was recently suggested for the pathogenesis of chronic gastritis elicited by *H. pylori *in humans [17,34,36].

In conclusion, we found that morphologically different *Helicobacter *species tend to differ in their abundance and regional distribution in the fundic mucosa. Free mixing of *Helicobacter *spp. was observed all along the length of gastric glands. The amounts of *H. bizzozeronii *were similar in superficial and base portions of the glands. Overall *H. bizzozeronii *was more abundant than *H. felis *in both intraluminal and intraparietal localization. *H. felis *was predominantly localized in the superficial portions of the gastric glands and was almost absent from the base. We also showed a true intracytoplasmic localization of both viable and degenerate *Helicobacter *organisms in parietal cells (within lysosomes and free in the cytoplasm), and rarely in macrophages in the lamina propria. The intraparietal and intramacrophagic presence of *Helicobacter *spp. are of interest in elucidating the mechanisms involved in the modulation of the immune response against gastric *Helicobacter *spp. in affected dogs. However, despite the high levels of colonization and ultrastructural findings, no clinical signs and only mild histological gastritis were observed, suggesting that immune tolerance might be involved in canine *Helicobacter *spp. infection.

## Competing interests

The authors declare that they have no competing interests.

## Authors' contributions

AL carried out the ultrastructural studies, and drafted the manuscript. IF participated in the ultrastructural studies. PC coordinated the ultrastructural studies and contributed to the interpretation of the ultrastructural data. ML performed part of the molecular genetic studies. KS performed part of the molecular genetic studies and the statistical analysis. ES participated in the design of the study and critically revised the manuscript. CR participated in the design of the study, carried out the histopathological studies and co-drafted the manuscript. All authors read and approved the final manuscript.

## References

[B1] Eaton KA Dewhirst FE Paster B Tzellas JN Coleman BE Paola J Sherding R Prevalence and varieties of *Helicobacter *species in dogs from random sources and pet dogs: animal and public health implications J Clin Microbiol 1996 34 3165 3170 8940465 10.1128/jcm.34.12.3165-3170.1996 PMC229476

[B2] Hänninen ML Happonen I Saari S Jalava K Culture and characteristics of *Helicobacter bizzozeronii*, a new canine gastric *Helicobacter *sp Int J Syst Bacteriol 1996 46 160 166 8573490 10.1099/00207713-46-1-160

[B3] Jalava K Kaartinen M Utriainen M Happonen I Hänninen ML *Helicobacter salomonis *sp. nov., a canine gastric *Helicobacter *sp. related to *Helicobacter felis *and *Helicobacter bizzozeronii* Int J Syst Bacteriol 1997 47 975 982 10.1099/00207713-47-4-975 9336895

[B4] Jalava K On SL Vandamme PA Happonen I Sukura A Hänninen ML Isolation and identification of *Helicobacter *spp. from canine and feline gastric mucosa Appl Environ Microbiol 1998 64 3998 4006 9758832 10.1128/aem.64.10.3998-4006.1998 PMC106591

[B5] Priestnall SL Wiinberg B Spohr A Neuhaus B Kuffer M Wiedmann M Simpson KW Evaluation of "*Helicobacter heilmannii*" subtypes in the gastric mucosas of cats and dogs J Clin Microbiol 2004 42 2144 2151 10.1128/JCM.42.5.2144-2151.2004 15131182 PMC404595

[B6] Van den Bulck K Decostere A Baele M Vandamme P Mast J Ducatelle R Haesebrouck F *Helicobacter cynogastricus *ssp. nov., isolated from the canine gastric mucosa Int J Syst Evol Microbiol 2006 56 1559 1564 10.1099/ijs.0.63860-0 16825630

[B7] Happonen I Saari S Castren L Tyni O Hänninen ML Westermarck E Occurrence and topographical mapping of gastric *Helicobacter*-like organisms and their association with histological changes in apparently healthy dogs and cats Zentralbl Veterinarmed A 1996 43 305 315 8779805 10.1111/j.1439-0442.1996.tb00457.x

[B8] Haesebrouck F Pasmans F Flahou B Chiers K Maele M Meyns T Decostere A Ducatelle R Gastric *Helicobacters *in domestic animals and nonhuman primates and their significance for human health Clin Microbiol Rev 2009 22 202 223 10.1128/CMR.00041-08 19366912 PMC2668234

[B9] Lockard VG Boler RK Ultrastructure of a spiraled microorganism in the gastric mucosa of dogs Am J Vet Res 1970 31 1453 1462 5449901

[B10] Salomon H Ueber das Spirillum des Saugetiermagens und sein Verhalten zu den Belegzellen Zentbl Bakteriol 1896 19 433 442

[B11] Weber AF Hasa O Sautter JH Some observations concerning the presence of spirilla in the fundic glands of dogs and cats Am J Vet Res 1958 19 677 680 13559622

[B12] Henry GA Long PH Burns JL Charbonneau DL Gastric spirillosis in Beagles Am J Vet Res 1987 48 831 836 3592386

[B13] Hermanns W Kregel K Breuer W Lechner J *Helicobacter*-like organisms: histopathological examination of gastric biopsies from dogs and cats J Comp Pathol 1995 112 307 318 10.1016/S0021-9975(05)80083-0 7560305

[B14] Wiinberg B Spohr A Dietz HH Egelund T Greiter-Wilke A McDonough SP Olsen J Priestnall S Chang YF Simpson KW Quantitative analysis of inflammatory and immune responses in dogs with gastritis and their relationship to *Helicobacter *spp. infection J Vet Intern Med 2005 19 4 14 15715041 10.1892/0891-6640(2005)19<4:qaoiai>2.0.co;2

[B15] Petersen AM Krogfelt KA *Helicobacter pylori*: an invading microorganism? A review FEMS Immunol Med Microbiol 2003 36 117 126 10.1016/S0928-8244(03)00020-8 12738380

[B16] Dubois A Borén T *Helicobacter pylori *is invasive and it may be a facultative intracellular organism Cell Microbiol 2007 9 1108 1116 10.1111/j.1462-5822.2007.00921.x 17388791 PMC1913845

[B17] Necchi V Candusso ME Tava F Luinetti O Ventura U Fiocca R Ricci V Solcia E Intracellular, intercellular, and stromal invasion of gastric mucosa, preneoplastic lesions, and cancer by *Helicobacter pylori* Gastroenterology 2007 132 1009 1023 10.1053/j.gastro.2007.01.049 17383424

[B18] Leib MS Duncan RB Ward DL Triple antimicrobial therapy and acid suppression in dogs with chronic vomiting and gastric *Helicobacter *spp J Vet Intern Med 2007 21 1185 1192 18196724 10.1892/06-135.1

[B19] Fox JG Dewhirst FE Shen Z Feng Y Taylor NS Paster BJ Ericson RL Lau CN Correa P Araya JC Roa I Hepatic *Helicobacter *species identified in bile and gallbladder tissue from Chileans with chronic cholecystitis Gastroenterology 1998 114 755 763 10.1016/S0016-5085(98)70589-X 9516396

[B20] De Groote D Haesebrouck F van Doorn LJ Vandamme P Ducatelle R Evaluation of a group-specific 16S ribosomal DNA-based PCR for detection of *Helicobacter bizzozeronii*, *Helicobacter felis*, and *Helicobacter salomonis *in fresh and paraffin-embedded gastric biopsy specimens J Clin Microbiol 2001 39 1197 1199 10.1128/JCM.39.3.1197-1199.2001 11230459 PMC87905

[B21] Neiger R Dieterich C Burnens A Waldvogel A Corthésy-Theulaz I Halter F Lauterburg B Schmassmann A Detection and prevalence of *Helicobacter *infection in pet cats J Clin Microbiol 1998 36 634 637 9508286 10.1128/jcm.36.3.634-637.1998 PMC104599

[B22] Recordati C Gualdi V Craven M Sala L Luini M Lanzoni A Rishniw M Simpson KW Scanziani E Spatial distribution of *Helicobacter *spp. in the gastrointestinal tract of dogs Helicobacter 2009 14 180 191 10.1111/j.1523-5378.2009.00674.x 19702848

[B23] Hsu SM Raine L Fanger H Use of avidin-biotin-peroxidase complex (ABC) in immunoperoxidase techniques: a comparison between ABC and unlabeled antibody (PAP) procedures J Histochem Cytochem 1981 29 577 580 6166661 10.1177/29.4.6166661

[B24] Lee A Hazell SL O'Rourke J Kouprach S Isolation of a spiral-shaped bacterium from the cat stomach Infect Immun 1988 56 2843 2850 3169989 10.1128/iai.56.11.2843-2850.1988 PMC259659

[B25] Simpson KW Strauss-Ayali D McDonough PL Chang YF Valentine BA Gastric function in dogs with naturally acquired gastric Helicobacter spp. infection J Vet Intern Med 1999 13 507 515 10587249 10.1892/0891-6640(1999)013<0507:gfidwn>2.3.co;2

[B26] Happonen I Linden J Saari S Karjalainen M Hänninen ML Jalava K Westermarck E Detection and effects of helicobacters in healthy dogs and dogs with signs of gastritis J Am Vet Med Assoc 1998 213 1767 1774 9861972

[B27] Lee A Krakowka S Fox JG Otto G Eaton KA Murphy JC Role of *Helicobacter felis *in chronic canine gastritis Vet Pathol 1992 29 487 494 10.1177/030098589202900601 1448894

[B28] De Bock M Decostere A Van den Bulck K Baele M Duchateau L Haesebrouck F Ducatelle R The inflammatory response in the mouse stomach to *Helicobacter bizzozeronii*, *Helicobacter salomonis *and two *Helicobacter felis *Strains J Comp Pathol 2005 133 83 91 10.1016/j.jcpa.2005.01.007 15949811

[B29] De Bock M D'Herde K Duchateau L Hellemans A Decostere A Haesebrouck F Ducatelle R The effect of *Helicobacter felis *and *Helicobacter bizzozeronii *on the gastric mucosa in Mongolian gerbils: a sequential pathological study J Comp Pathol 2006 135 226 236 10.1016/j.jcpa.2006.08.003 17069831

[B30] De Bock M Decostere A Hellemans A Haesebrouck F Ducatelle R *Helicobacter felis *and *Helicobacter bizzozeronii *induce gastric parietal cell loss in Mongolian gerbils Microbes Infect 2006 8 503 510 10.1016/j.micinf.2005.08.003 16311055

[B31] Noach LA Rolf TM Tytgat GN Electron microscopic study of association between *Helicobacter pylori *and gastric and duodenal mucosa J Clin Pathol 1994 47 699 704 10.1136/jcp.47.8.699 7962619 PMC502139

[B32] Heilmann KL Borchard F Gastritis due to spiral shaped bacteria other than *Helicobacter pylori*: clinical, histological, and ultrastructural findings Gut 1991 32 137 140 10.1136/gut.32.2.137 1864530 PMC1378794

[B33] Scanziani E Simpson KW Monestiroli S Soldati S Strauss-Ayali D Del Piero F Histological and immunohistochemical detection of different *Helicobacter *specie in the gastric mucosa of cats J Vet Diagn Invest 2001 13 3 12 11243359 10.1177/104063870101300102

[B34] Ito T Kobayashi D Uchida K Takemura T Nagaoka S Kobayashi I Yokoyama T Ishige I Ishige Y Ishida N Furukawa A Muraoka H Ikeda S Sekine M Ando N Suzuki Y Yamada T Suzuki T Eishi Y *Helicobacter pylori *invades the gastric mucosa and translocates to the gastric lymph nodes Lab Invest 2008 88 664 681 10.1038/labinvest.2008.33 18475258

[B35] Simpson KW McDonough PL Strauss-Ayali D Chang YF Harpending P Valentine BA *Helicobacter felis *infection in dogs: effect on gastric structure and function Vet Pathol 1999 36 237 248 10.1354/vp.36-3-237 10332832

[B36] Ye G Barrera C Fan X Gourley WK Crowe SE Ernst PB Reyes VE Expression of B7-1 and B7-2 costimulatory molecules by human gastric epithelial cells. Potential role in CD4+ T cell activation during *Helicobacter pylori *infection J Clin Invest 1997 99 1628 1636 10.1172/JCI119325 9120006 PMC507982

